# Patient Inspiratory Maneuver Performance; Peak Lungpower, Acceleration and Volume

**DOI:** 10.1089/jamp.2019.1575

**Published:** 2020-12-02

**Authors:** Jussi Haikarainen, Mikko Vahteristo, Satu Lähelmä, Ville Vartiainen, Leo Pekka Malmberg

**Affiliations:** ^1^ Orion Corporation Orion Pharma, Espoo, Finland.; ^2^ Allergology, University of Helsinki and Helsinki University Hospital, Helsinki, Finland.

**Keywords:** airflow, airflow resistance, asthma, COPD, dry powder inhalers, lungpower

## Abstract

***Background:*** Use of drug delivery devices between nebulizers, dry powder inhalers (DPIs), or metered dose inhalers (MDIs), for treating patients with asthma and chronic obstructive pulmonary disease (COPD), is based on patients' capability of coordinating the inhalation maneuver and achieving sufficient airflow. There are limited data available with regard to how patients meet the requirements of successful inhalation performance, and how the concept of inspiratory lungpower could be applied. The aim of this work was to study the patient inspiratory airflow profile performance in large data sets. We analyzed how the Kamin-Haidl inhalation criteria were met by patients with DPIs such as Easyhaler for combination therapy (EH-combi), Easyhaler for monotherapy (EH-mono), Diskus, and Turbuhaler (TH), and applied peak lungpower instead of peak inspiratory flow rate as an indicator of patient performance.

***Materials and Methods:*** Data sets gathered in two previous studies for DPIs, that is, EH-combi, EH-mono, Diskus, and TH, were used to analyze how inspiratory lungpower representing inspiratory muscle power, flow acceleration, and volume after peak met the inhalation criteria. The measured patient airflow profiles through inhalers were assessed for patients with asthma or COPD.

***Results:*** Based on the Kamin-Haidl inhalation criteria, successful inhalation requirements were met with EH-combi in 96.1% and with EH-mono in 92.6% of patients. The success rates were 89.5% and 84.6% with Diskus and TH, respectively, (*p* < 0.0001 between devices). In patients with asthma or COPD, the mean lungpower was 7.51 and 6.15 W for EH-combi, 8.79 and 6.88 W for EH-mono, 7.18 and 4.36 W for Diskus, and 9.65 and 6.86 W for TH, respectively, when patients followed the manufacturer's written instructions.

***Conclusions:*** Lungpower applied to the Kamin-Haidl inhalation criteria concept could be an applicable method for reviewing patient performance for different DPIs despite DPIs' characteristic differences in airflow resistance. In light of these results, DPIs provide a feasible treatment option for a large majority of respiratory patients.

## Introduction

A degree of misinterpretation relates to the patient inspiratory flow rate with respect to inhalation capability. The discussion on inspiratory effort has been based mostly on achieved peak inspiratory flow (PIF) alone, which may lead to erroneous conclusions unless the inhaler's airflow resistance is also considered. The PIF does not translate well between inhalers with different airflow resistances, as lower the resistance, the higher the flow rate will be. The concept of lungpower, defined in equation [3], takes into account both airflow resistance and airflow and could provide better means for representing the patient inspiratory muscle power.^(1–6)^

In the 1960s, the respiratory muscle strength determination method was developed by Black and Hyatt and defined as maximum inspiratory pressure (MIP) when measured at fixed airflow resistance.^(4)^ The MIP measurement was carried out inhaling and exhaling through a cylindrical tube with a small opening of ∅2 mm × 15 mm, reducing the excessive mouth pressure compared with closed volume^(4,5)^ and measuring the generated mouth pressure. The small hole in this setup is analogous to airflow resistance of an inhaler. MIP describing inspiratory muscle strength was defined as pressure with the given fixed airflow resistance; later, for example, 100 cm/H_2_O at 220 mL/s flow ( = 0.24 √kPa min/L) by Enright.^(5)^ This MIP measurement procedure has been in use for a long time, with minor modifications. For example, de Köning used a modified setup using a resistance of 0.039 √kPa min/L.^(7)^ By equation [1], PIF has a negative correlation to inhaler resistance, whereas pressure drop has a positive correlation.

Kamin et al. defined criteria for assessing successful inhalation maneuver.^(8,9)^ The work was continued by Haidl et al. by extensive literature review looking at different inhalation devices, *in vitro* evidence, and most-suited inhalation maneuver.^(10)^ The Kamin-Haidl criterion for PIF is 30 L/min for Easyhaler for combination therapy (EH-combi), Easyhaler for monotherapy (EH-mono), and Diskus.^(9–16)^ PIF recommendation for Turbuhaler (TH) is higher (60 L/min)^(8,9,17,18)^ than for many other dry powder inhalers (DPIs) based on the findings of TH's high airflow dependency in low-to-medium airflows.^(10,19)^ The acceleration criterion^(8–10)^ for all these devices is 0.7 L/s^2^ and the requirement for inhalation volume is 500 mL after reaching the peak flow rate. These Kamin-Haidl criteria are represented in Table 1. Haidl's work was further continued by Pohlmann et al. using the concept of lungpower and examined the minimum peak lungpower criterion with a small group of subjects for inhalers based on Haidl's values.^(8–10,19)^

**Table 1.** Kamin-Haidl Inhalation Criteria

Kamin-Haidl inhalation criteria	
PIF	≥30 L/min^a^
Inhalation acceleration	≥0.7 L/s^2^
Inhalation volume	≥500 mL after PIF

^a^ TH 60 L/min^10^

PIF, peak inspiratory flow; TH, Turbuhaler.

Dunbar et al.^(20)^ investigated power using 1, 2, and 3 W power in his *in vitro* performance work. They showed on two different devices out of three that DPIs can deliver drugs already at a power of 1 W. Dunbar et al. concluded that lungpower could be an applicable method studying inhalers with different resistances in a comparable manner, but it would be important to investigate applicable power levels on clinical setting.

We used Kamin-Haidl inhalation criteria for inhalation performance assessment. We replaced PIF by using conversion to lungpower according to equation [3] and included inhalation acceleration and volume after the peak determination. We also investigated how peak lungpower relates to the achieved airflows and pressure drops using different inhalers. We analyzed patient peak lungpower data based on PIF-studies that were carried out for four different DPIs.^(21,22)^

## Materials and Methods

The PIF rate studies for this work have been previously reported in detail by Malmberg et al.^(21)^ and Jõgi et al.^(22)^ Briefly, the study population included children, adults, and elderly patients with asthma or chronic obstructive pulmonary disease (COPD).

There were 187 patients included in the study by Malmberg et al. and 227 patients in the study by Jõgi et al. From the studies, 17 patients were excluded from per protocol analyses. In addition, 14 subjects younger than 6 years from the study by Jõgi et al. were excluded further from the analyses. Thus, the total number of subjects in this study is 383. Out of these, 287 were asthmatics and 96 were patients with COPD. The inhalation profile data were available for 363, 202, 200, and 162 patients for EH-combi, EH-mono, Diskus, and TH, respectively.

In these studies, the lactose placebo-filled commercial DPI products of budesonide/formoterol Easyhaler (EH-combi) (Orion Pharma, Finland), budesonide Easyhaler (EH-mono) (Orion Pharma), salmeterol/fluticasone Easyhaler (EH-combi) (Orion Pharma), salmeterol/fluticasone Diskus (Diskus) (GSK Pharma, United Kingdom), and budesonide/formoterol TH (AstraZeneca, United Kingdom) were used.

Inhaler airflow resistances were 0.036 √kPa min/L for EH-combi, 0.044 for EH-mono, 0.027 for Diskus, and 0.032 for TH (TH M3-version).^(21,22)^

The inhalation flow profile measurements were conducted according to manufacturer's written instructions (patient information leaflets) to conduct the inhalation maneuver as they are taught in real-life use. The PIFs and volumes through inhalers were identified for patients with asthma or COPD.

Inhalation maneuver instructions for the study by Malmberg et al. and the study by Jõgi et al. are provided in Table 2.

**Table 2.** The Applied Inhalation Maneuver for EH-Combi, EH-Mono, Diskus, and Turbuhaler

Device	Device-specific inhalation maneuver instruction
EH-combi	Take a strong and deep breath through the Easyhaler.
EH-mono	Take a strong and deep breath through the Easyhaler.
Diskus	Breathe in steadily and deeply through the Diskus, not through your nose.
TH	Breathe in as deeply and as hard as you can through your mouth.

EH-combi, Easyhaler for combination therapy; EH-mono, Easyhaler for monotherapy.

### Inhalation flow profile

Complete inspiratory flow profiles through inhalers were recorded using pneumotachograph (Spiromaster MX; Medikro Ltd., Kuopio, Finland), including pressure drops, and inspiratory volumes. For the purposes of this study, the complete inspiratory flow data sets that were measured at 100 Hz (10 ms) interval were reanalyzed. Further analysis on the flow profile data was conducted for PIF rate time point and inhaled volume beyond the PIF.

### Kamin-Haidl inhalation criteria and lungpower

The Kamin-Haidl^(9,10)^ inhalation criteria were used for inhalation performance assessment. PIF was converted to lungpower by applying equation [3].^(19)^ Inhalation acceleration and volume after the peak determination were used as presented by the original authors. All three criteria were to be met during the same inhalation maneuver for a successful attempt.

Clark & Hollingworth^(23)^ provide equation [1] for the pressure drop within the inhaler system for turbulent flow.

[1] √ΔP=Q × R (ΔP for pressure drop,Q for airflow and R for airflow resistance)

Harris applied the concept of air watts^(2,3)^ with respect to equation [2] for inhaler's vacuum power.

[2] P=Q×ΔP(PforPower,QforairflowandΔPforpressuredrop)

Equation [3] was introduced by Dunbar et al., which can be originated from [1] and [2], and it gives lungpower as a function of airflow and resistance.^(20,24)^

[3] $$P=Q^{3}\cdot R^{2}\left(PforPower,QforairflowandRforairflowresistance\right)$$

With the commonly used units in inhalation literature for *Q* (L/min) and *R* (√kPa min/L) unit conversion into SI-base units of m/s and √kPa s/L this is written as follows:

[4] $$P={\left(Q∕60\right)}^{3}\cdot {\left(60\cdot R\right)}^{2}$$

When converting the PIF from Kamin-Haidl criteria to lungpower according to equation [3] at a recommended flow of 30 L/min, the peak lungpower acceptance criteria were 0.58 W for EH-combi, 0.87 W for EH-mono, and 0.33 W for Diskus. The TH lungpower threshold value is 3.69 W based on 0.032 √kPa min/L resistance at a recommended 60 L/min PIF.

### Peak time, airflow acceleration, and volume after peak

Peak time is the time point in the inspiratory flow profile where the maximum inspiratory flow rate occurs. Flow acceleration [or flow increase rate (FIR)] was calculated as the numerical derivative of the flow rate, as described in equation [5].

[5] FIR=ΔQ∕Δt

For the acceleration, we used 10 L/min as lower end and 80% of the maximum as upper limit for the derivation. In literature, 20% of the maximum is often used as a lower limit.^(2,7)^ However, we consider our approach valid since it correctly identified the linear region of the patient inhalation profile curve. Acceptance criteria according to Kamin-Haidl were for acceleration 0.7 m/s^2^ and for volume 500 mL after the peak.^(9,10)^

### Inhalation work

Inhalation work was calculated by determining lungpower area under the curve for the duration of the inhalation maneuver according to equation [6]. The airflow is measured with the mentioned Spiromaster MX pneumotachograph, and lungpower calculated according to equation [4] at 100 Hz frequency. Equation [6] was discretized using trapezoidal rule.

[6] E=∫Pdt

### Statistical methods

Pairwise comparisons of inhalers according to the Kamin-Haidl criteria were performed using McNemar's test. All descriptive statistics and statistical comparisons were performed with SAS^®^ ver. 9.4 (SAS Institute, Inc., Cary, NC).

## Results

### Inhalation flow profile

The Malmberg et al. and Jõgi et al. inhalation profile characteristics were compiled from the studies and are presented in Table 3.

**Table 3.** Mean Peak Inspiratory Flow (L/Min) and Inspiratory Volume Through EH-Combi, EH-Mono, Diskus, and Turbuhaler Inhalers in Patients with Asthma or Chronic Obstructive Pulmonary Disease (Per-Protocol Data Set)^(21,22)^

	Patients with asthma			Patients with COPD		
	n	PIF (std)	Volume (std)	n	PIF (std)	Volume (std)
EH-combi	287	66.7 (13.4)	1.9 (0.7)	96	61.9 (13.2)	1.8 (0.7)
EH-mono	150	60.9 (12.0)	2.0 (0.7)	52	58.2 (8.6)	1.9 (0.6)
Diskus	150	76.6 (20.0)	2.3 (0.9)	52	65.1 (19.0)	2.2 (0.7)
TH	137	79.4 (14.4)	1.9 (0.7)	44	72.1 (15.1)	1.8 (0.6)

*N* reflects the obtained PIF and volume values in the studies and thus differs slightly from the obtained number of inhalation profiles.

COPD, chronic obstructive pulmonary disease.

For performance assessment, we analyzed the inhalation profiles for lungpower, the timing when the peak flow rate was achieved (Tmax), acceleration rate, and inhalation work. The results are provided in Table 4.

**Table 4.** Patient Inhalation Parameters for EH-Combi, EH-Mono, Diskus, and Turbuhaler

Disease group	Treatment (resistance)	Peak lung power (W)**Mean (std)	Peak lung power 10th percentile	Peak lung power 90th percentile	Tmax (s)**Mean (std)	Acceleration (L/s^2^)**Mean (std)	Acceleration 10th percentile	Acceleration 90th percentile	Work (J) mean (std)
Asthma	EH-combi (0.036)	7.51 (4.73)	2.77	13.08	0.57 (0.30)	4.27 (2.67)	1.45	7.06	8.43 (7.97)
	EH-mono (0.044)	8.79 (7.24)	3.18	13.61	0.60 (0.30)	3.84 (2.47)	0.98	6.86	11.43 (14.78)
	DISKUS (0.027)	7.18 (7.14)	1.61	13.35	0.88 (0.62)	3.78 (3.32)	0.86	7.50	7.77 (9.34)
	TH (0.032)	9.65 (4.92)	3.69	15.67	0.48 (0.20)	5.42 (2.62)	2.42	9.33	8.93 (6.12)
COPD	EH-combi (0.036)	6.15 (3.42)	2.21	11.74	0.47 (0.14)	4.16 (2.10)	1.93	6.51	6.55 (4.34)
	EH-mono (0.044)	6.88 (2.93)	3.84	11.16	0.54 (0.22)	3.66 (1.80)	1.65	6.39	8.23 (4.12)
	DISKUS (0.027)	4.36 (3.78)	1.29	8.51	0.90 (0.60)	2.85 (2.31)	0.77	5.49	4.85 (3.26)
	TH (0.032)	6.86 (3.78)	1.96	11.47	0.48 (0.20)	5.17 (2.91)	2.19	7.30	6.27 (4.20)

### Kamin-Haidl inhalation criteria and lungpower

**FIG. 1.**
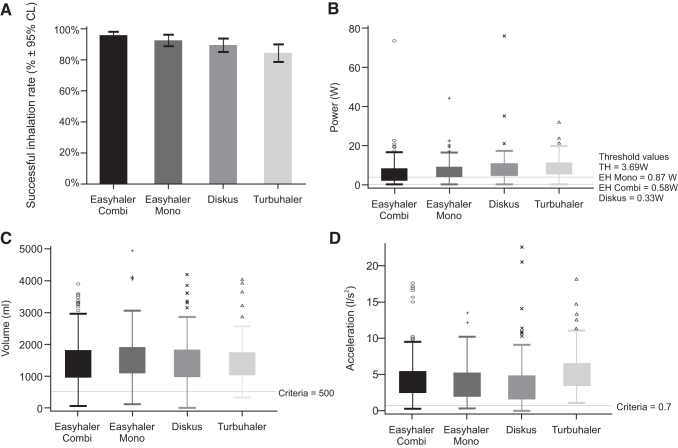
Patient inhalation performance for EH-combi, EH-mono, Diskus, and TH. **(A)** Successful inhalation rate (Kamin-Haidl) for EH-combi, EH-mono, Diskus, and TH. **(B)** Peak lungpower for EH-combi, EH-mono, Diskus, and TH. **(C)** Inhalation volume after peak for EH-combi, EH-mono, Diskus, and TH (the horizontal line in figures **B–D** represents success criterion). **(D)** Inhalation acceleration for EH-combi, EH-mono, Diskus, and TH. EH-combi, Easyhaler for combination therapy; EH-mono, Easyhaler for monotherapy; TH, Turbuhaler.

For assessing the successful inhalation rates for different devices, we used complete flow profile data, where in addition to lungpower and acceleration rate, we checked that 500 mL inhalation volume after the PIF was reached. The analyses of the different parameters for Kamin-Haidl inhalation criteria are summarized in Table 5 and Figure 1A–D.

**Table 5.** Successful Inhalation Performance Attempts and Percentage with Different Dry Powder Inhalers

Device	Patients who meet device-specific peak lungpower criteria	Acceleration >0.7 L/s^2^	Volume after PIF >500 mL	Successful inhalation rate (Kamin-Haidl)* n *(%)
EH-combi (*n* 363)	360 (99.2%)	357 (98.4%)	353 (97.3%)	349 (96.1%)
EH-mono (*n* 202)	199 (98.5%)	194 (96.0%)	192 (95.1%)	187 (92.6%)
Diskus (*n* 200)	198 (99.0%)	185 (92.5%)	190 (95.0%)	179 (89.5%)
TH (*n* 162)	139 (85.8%)	162 (100%)	158 (97.5%)	137 (84.6%)

For calculation of the lungpower, the pressure drops were measured first as described and values used for calculating PIF according to equation [1]. The pressure drop results are compiled in Table 6. For the EH-combi, the mean peak pressure drop for patients with asthma was 6.15 kPa (95% confidence interval [CI]: 5.86–6.45), EH-mono 7.75 kPa (7.17–8.33), Diskus 4.78 (4.34–5.22), and TH 6.80 kPa (6.40–7.21), respectively. For patients with COPD, the mean peak value for EH-combi was 5.41 kPa (4.96–5.85), for EH-mono 6.76 kPa (6.22–7.30), for Diskus 3.43 kPa (2.89–3.98), and for TH 5.37 (4.65–6.09), respectively. The 10th and 90th percentile peak pressure values followed the same inhaler rank order for the two different patient groups similar to the means. The range for 10th percentile was from 1.89 to 4.11 kPa for patients with asthma and from 1.63 to 4.65 kPa for patients with COPD. In turn, 90th percentile values were from 9.28 to 10.87 kPa for patients with asthma and from 5.74 to 9.54 kPa for patients with COPD.

**Table 6.** Measured Pressure Drop Characteristics for Patients with Asthma and Chronic Obstructive Pulmonary Disease

Disease group	Treatment (*n*)	Mean pressure drop (kPa)	95% lower CL for mean	95% upper CL for mean	10% percentile for pressure drop	90% percentile for pressure drop
Asthma	EH-combi (277)	6.15	5.86	6.45	3.31	9.28
	EH-mono (150)	7.75	7.17	8.33	4.11	10.87
	Diskus (149)	4.78	4.34	5.22	1.89	7.77
	TH (127)	6.80	6.40	7.21	3.65	9.65
COPD	EH-combi (86)	5.41	4.96	5.85	2.83	8.68
	EH-mono (52)	6.76	6.22	7.30	4.65	9.54
	Diskus (51)	3.43	2.89	3.98	1.63	5.74
	TH (35)	5.37	4.65	6.09	2.44	7.84

CL, confidence limit.

The peak lungpower mean values with the four devices were between 7.18 and 9.65 W for patients with asthma, and from 4.36 to 6.88 W for patients with COPD. For EH-combi, the mean peak value was 7.51 W (95% CI: 6.95–8.07) for asthmatics and 6.15 W (5.42–6.88) for patients with COPD. For EH-mono, the measured peak lungpower mean was 8.79 W (7.63–10.0) for patients with asthma and 6.88 W (6.06–7.69) for patients with COPD. The lowest values were obtained with Diskus for patients with asthma, 7.18 W (6.02–8.33), and patients with COPD produced 4.36 W (3.302–5.43) of peak lungpower, whereas TH results were 9.65 W (8.79–10.52) for asthmatics and 6.86 W (5.56–8.16) for patients with COPD. The peak lungpower results are illustrated in Figure 1B.

Kamin-Haidl inhalation criteria were met for 96.1% (95% CI: 94.2–98.1) of EH-combi, 92.6% (88.9–96.2) of EH-mono, 89.5% (85.2–93.8) of Diskus users, and 84.6% (78.9–90.2) of TH. The performances on different inhalers are shown in Figure 1A.

### Peak time, airflow acceleration, and volume after the peak

The mean peak timings (Tmax) were for patients with asthma with EH-combi 0.57 second (95% CI: 0.53–0.60), EH-mono 0.60 second (0.55–0.65), Diskus 0.88 second (0.78–0.98), and TH 0.48 second (0.45–0.52), respectively. Correspondingly, for patients with COPD EH-combi, these were 0.47 second (0.44–0.50), EH-mono 0.54 second (0.48–0.60), Diskus 0.90 second (0.73–1.07), and TH 0.48 second (0.41–0.55), respectively.

The mean acceleration for EH-combi was 4.27 L/s^2^ (95% CI: 3.96–4.59) and 4.16 L/s^2^ (3.71–4.61) for asthmatics and COPD patients, respectively. EH-mono had very similar mean results between asthmatics and COPD patients, 3.84 L/s^2^ (3.45–4.24) versus 3.66 L/s^2^ (3.16–4.16), while the 10th percentile differed slightly, being 0.98 versus1.65 L/s^2^. Acceleration on TH measured similar values 5.42 L/s^2^ (4.96–5.88) and 5.17 L/s^2^ (4.18–6.17) for both patient groups. Results for COPD patients with Diskus were lowest 2.85 L/s^2^ (2.20–3.50), and 0.77 L/s^2^ by 10th percentile. Patients with asthma reached mean value of 3.78 L/s^2^ (3.24–4.32) with Diskus. The patient accelerations are shown in Figure 1D.

For patients with asthma, mean volume after the peak was for EH-combi 1.47 L (95% CI: 1.39–1.55), EH-mono 1.55 L (1.43–1.67), Diskus 1.47 L (1.35–1.60), and for TH 1.47 L (1.36–1.59). In turn, for patients with COPD, the results were 1.44 L (1.32–1.56), EH-mono 1.50 L (1.35–1.64), Diskus 1.47 L (1.31–1.63), and for TH 1.26 L (1.11–1.41), respectively. Figure 1C illustrates patient volume after the peak performance.

### Inhalation work

The inhalation work was relatively similar among patients with asthma having mean inhalation work for EH-combi 8.43 J (95% CI: 7.49–9.37), Diskus 7.77 J (6.26–9.29), and TH 8.93 J (7.86–10.01). EH-mono measured higher, 11.43 J (9.04–13.81). For the COPD patients, EH-combi and TH were similar with measured work of 6.55 J (5.62–7.49) and 6.27 J (4.83–7.72), respectively. EH-mono was measured at 8.23 J (7.08–9.37) and Diskus at 4.85 J (3.93–5.77).

### Relevance of lungpower in airflow dependency testing

We observed that patients seldom (in total, 11 out of 383 patients) exceeded the peak lungpower of 20 W. Consequently, the flow rates had device-specific area of relevance for peak lungpowers of 0–20 W. Table 7 provides calculations for EH-combi, EH-mono, TH, and Diskus with the flow rate-specific lungpower values. These power values provide patient inspiratory effort comparability from one device to another with a given airflow.

**Table 7.** Calculated Inhalation Power of Tested Inhalers with Fixed Airflows

	Resistance (√kPa min/L)	Airflow (L/min)									
		30	40	50	60	70	80	90	100	110	120
EH-mono	0.044	0.87	2.07	4.03	6.97	11.07	16.52	23.52	32.27	42.95	55.76
EH-combi	0.036	0.58	1.38	2.70	4.67	7.41	11.06	15.75	21.60	28.75	37.32
TH	0.032	0.46	1.09	2.13	3.69	5.85	8.74	12.44	17.07	22.72	29.49
Diskus	0.027	0.33	0.78	1.52	2.62	4.17	6.22	8.86	12.15	16.17	21.00

Table 7 shows that the flow rates would be limited by patients' lungpower with higher resistance devices only at very high flow rates. Airflow of 90 L/min flow already represents 23.5 W of lungpower with high resistance EH-mono. This is nearly three times the lungpower than that with medium resistance Diskus (8.86 W) with the same airflow. We observed that applying fixed airflows of, for example, common 30, 60, and 90 L/min do not indicate the same patient population groups at 60 and 90 L/min regarding inspiratory muscle strength with devices of different airflow resistances.

## Discussion

The inhaler prescription routine follows the principles of first selecting the appropriate active substance and dose for the patient. The device selection (nebulizer, metered dose inhaler [MDI], DPI) for the patient can be based on the patient's capability to coordinate the maneuver and inhalation efficiency.^(17)^ Very young children or patients, who cannot coordinate their actuation/inhalation maneuver or do not achieve the recommended PIF threshold via a DPI (most often 30 L/min) could be recommended to use a nebulizer or MDI. DPIs are an alternative for patients who achieve the threshold flow as they resolve the coordination requirements related with MDIs.^(17)^ Inhalers can provide different deagglomeration efficacy at same airflow if device's resistance and design are different.^(17)^ In some inhalers such as EH, the particles are locally accelerated utilizing the Bernoulli principle. The Bernoulli law holds also for gasses as long as the velocity is sufficiently low (<1/3 speed of sound) for them to be treated as incompressible. The particle wall collisions are intended to occur when the kinetic energy of the particles is at the maximum leading to high fine particle fraction (FPF). When deagglomeration has taken place, the particles are slowed down again in an expanded flow tube and further in the mouth. In EH, the airflow resistance results from the flow constriction and is therefore an important feature of the device. It does not increase or decrease the total energy that is fed into the system by the patient and that is provided for particle dispersion.

For TH, we noted that the higher minimum peak lungpower requirement due to higher minimum flow requirement^(8,9,17,18)^ has led to special emphasis on instruction language: “breathe in as deeply and as hard as you can through your mouth.” This instruction update was studied and results were reported by Persson et al.^(25)^ TH instruction language encourages the patients to take their best effort in inhalation maneuver and that can be seen from slightly higher lungpower recordings than for other devices. According to Persson et al., this increased PIF in average 20% compared with the old instruction “take a deep inhalation from the inhaler.”^(25)^ In turn, the inhalation instruction language for Diskus is similar to many MDIs and states that patient should “breathe in steadily and deeply.” It was discussed by Broeders et al.^(26)^ that the wording may compromise the effort by the patient, which converts to lower than expected inhalation acceleration. Whether the same inhalation instructions for all devices would measure the same lungpower regardless of resistance is an area of further studies.

For patients with asthma, the measured lungpower mean was from 6 W to 10 W, and from 4 to 7 W for patients with COPD. The lowest values were recorded for Diskus, 4 W, by patients with COPD. For DPI usage, these observed lungpowers are plentiful in comparison with minimum criteria of Pohlmann.^(19)^ For EH-combi, the threshold according to formula [3] would be 0.6 W, and Pohlmann had determined the threshold for EH-mono as 1.2 W, TH 4.4 W, and Diskus 0.3 W.^(16)^ Therefore, we believe that patient peak lungpower does not limit the use of DPIs as the minimum peak lungpower criteria were met to high degree by both EH and Diskus users and only with TH patients, capability was slightly compromised. For verifying our results, we back-calculated Enright et al.'s^(5)^ 1994 work; Respiratory Muscle Strength in the Elderly (4443 participants, 65 years and older, men and female), MIP. We found that indeed the mean peak lungpower levels are 7.15–16.10 W for men and 3.65–7.94 W for women. This Enright study uses a setup that has a resistance of 0.24 √kPa min/L, nearly 10-fold of medium resistance inhaler and still the results in power make sense in comparison with our findings.

Based on the measured Tmax,^(25)^ acceleration, and comparison to literature, for example, Azouz et al.^(27)^ (who encouraged patients to inhale as they normally would) provide further evidence that the device's instruction language may have significance on patient's performance.^(25,26)^ This may explain the contradictionary results of observing the longest Tmax and lowest acceleration with Diskus that has lowest resistance opposite to learnings in the literature.^(6,27)^

The volume after the peak was very similar between all tested devices and corresponds to the knowledge that resistance does not alter patients' inhalation volume, which is restricted by the lung volume.

Inhalation work is defined by equation [6] and corresponds to the area under the curve of the lungpower versus time curve.^(19)^ Therefore, we see the highest performance with the patients who have the highest lung capacity in terms of power and volume, that is, the patients with asthma in the study by Malmberg et al. and study by Jõgi et al. The lower the resistance the higher the peak inhalation flow, but shorter the duration. As expected from the literature, the inhalation work was found to be reasonably similar between the inhalers despite their resistances.^(28)^ It is likely that this parameter is also affected by the provided patient instructions.

Kamin-Haidl inhalation criteria were met for 96% of EH-combi, 93% of EH-mono, 90% of Diskus, and 85% of TH users. In a study by Malmberg et al., approximately 14% of TH users struggled in producing sufficient lungpower. Persson et al. had earlier reported 67% TH users meeting 60 L/min with a device having resistance near the commercial products.^(25)^ Unexpectedly, meeting the required acceleration was most challenging for Diskus users in our study. According to literature,^(6)^ it should be the opposite for lower resistance devices. There was no substantial difference between the devices in meeting the volume after the peak criteria.

Results of our study show no compromise in lungpower generation nor airflow acceleration for the highest resistance device EH-mono or medium resistance Diskus having a threshold of 30 L/min. Similar to Kamin-Haidl criteria, a combination of inspiratory effort, acceleration, and inhalation volume was proposed also by Azouz and Chrystyn.^(29)^

In literature, a higher inhaler device resistance is considered to improve lung deposition.^(7,18)^ The considered benefits include reduction of particle speed and thus reduced oropharyngeal deposition, enabling higher lung deposition.^(7,18)^ In addition, de Köning suggested that a higher resistance device uses a smaller fraction (10% vs. 30%) of the lung volume, achieving sufficient acceleration for drug aerosolization. Therefore, excess lung volume provides extended inhalation duration that enables better fine drug particle transport into the targeted lung regions.^(7)^ According to a study in literature, a great majority of healthy volunteers (82%) have found resistances of 0.021–0.047 √kPa min/L acceptable range.^(28)^

Clark et al. discussed in favor of using inspiratory pressure instead of inspiratory flow rate, which they state being misleading in inhaler selection.^(30)^ By nature, when the resistance increases, PIF decreases and pressure drop increases according to equation [1]. This effect is shown in the study by Azouz et al. and Clark et al.^(27,30)^ We believe that the physical process of patient inspiratory muscle effort is described by lungpower and therefore remains constant for a specific patient regardless of inhaler resistance. Peak lungpower means shown in Figure 1B are fairly similar regardless of the device as expected if it describes the patient capability. Achieved pressure drops, however, show marked differences between the devices in Table 6. This is highlighted by the lack of overlap of CIs between the means of peak pressure drops.

This can be illustrated further by comparison at, for example, constant 10 kPa peak pressure drop, which is sometimes considered close to top range of human performance. Achieving 10 kPa peak pressure drop for high resistance EH-mono requires just 11.98 W, for medium to high resistance EH-combi it requires 14.64 W, and for medium resistance TH and Diskus 16.42 W and 19.52 W, respectively. These calculation results suggest that generating the same peak pressure drops for different inhaler devices would not represent equal inspiratory effort and would therefore describe different patient populations when applied to different inhalers.

Our results, illustrated in Table 7, indicate that at 60 and 90 L/min, the applied lungpower would differ drastically. The fixed values of 30, 60, and 90 L/min airflows for *in vitro* studies are therefore comparable only for devices of similar resistances. We believe that the most adequate method for *in vitro* flow rate investigations is to identify 10th, mean, and 90th patient percentile flow rates (or PIFs or lungpowers) for each device and patient group separately. Malmberg and Jõgi have done so in their studies, which were based on European Union's (EU's) Orally inhaled products' guideline.^(21,22)^ Concerning the product performance at different air flow percentiles, these studies reported similar fine particle airflow dependency performance and delivered dose performance for the EH-combi and TH or Diskus within 10th to 90th percentile airflows.^(21,22)^

In real life, patient lungpower measurement could be performed using device recording PIF or peak pressure drop, acceleration, and volume after the peak, such as a tailored spirometer. With known resistance, PIF or pressure drop can be converted to lungpower using equation [3]. We believe this data set provides valuable inhalation characterization data and is a helpful tool for understanding the lungpower concept, and peak lungpower distribution in intended patient groups. It also explores the usefulness of Kamin-Haidl inhalation criteria for assessing successful inhalation by patients.

## Conclusions

It has occasionally been questioned whether higher resistance inhaler devices would be more difficult to use for the patient. In this study, the patients performed well in lungpower generation, airflow acceleration, and inhalation volume after the peak. These results indicate that inhaler device resistance within the typical range of DPI resistances (in this study 0.027–0.044 √kPa min/L) does not limit the use of DPIs among typical patients with asthma or COPD.

Based on inhalation performance of the patients in our data set, we consider Kamin-Haidl criteria incorporated with peak lungpower as a potential option for investigating patient performance and confirming the suitable inhaler type for the patient. Peak lungpower is a patient-specific characteristic subject to the patients' use of their inspiratory muscles. Resistance is a device-specific fixed parameter, and the generated flow rate or pressure drop is an interplay of both inspiratory muscle use and resistance, as per equation [1]. This inspiratory effort is previously reported,^(25)^ neither to correlate strongly with the clinical condition nor to the peak expiratory flow, but rather the muscular strength of the patient. Further studies with harmonized inhalation instructions are required to show whether lungpower would be independent of the used inhaler. Reportedly, even severe asthmatics can produce almost normal inhalations through all tested devices.^(25)^

The use of Kamin-Haidl criteria could be further helped by developing suitable analytical hand tools for clinicians that provide lungpower outcome directly as a measurable.

## Notes

Reviewed by:

Jeffry Weers

Andy Clark
